# Diagnostic accuracy of the rapid urine lipoarabinomannan test for pulmonary tuberculosis among HIV-infected adults in Ghana–findings from the DETECT HIV-TB study

**DOI:** 10.1186/s12879-015-1151-1

**Published:** 2015-10-01

**Authors:** Stephanie Bjerrum, Ernest Kenu, Margaret Lartey, Mercy Jemina Newman, Kennedy Kwasi Addo, Aase Bengaard Andersen, Isik Somuncu Johansen

**Affiliations:** Department of Infectious Diseases, Odense University Hospital, Odense, Denmark; Institute of Clinical Research, University of Southern Denmark, Odense, Denmark; Fevers Unit, Korle-Bu Teaching Hospital, Accra, Ghana; Department of Medicine, University of Ghana Medical and Dental School, Accra, Ghana; Department of Medical Microbiology, School of Biomedical and Allied Sciences, College of Health Sciences, University of Ghana, Accra, Ghana; Department of Bacteriology, Noguchi Memorial Institute for Medical Research, University of Ghana, Accra, Ghana

**Keywords:** Tuberculosis, HIV, Lipoarabinomannan, Urine, Diagnosis, Africa, Ghana

## Abstract

**Background:**

Rapid diagnostic tests are urgently needed to mitigate HIV-associated tuberculosis (TB) mortality. We evaluated diagnostic accuracy of the rapid urine lipoarabinomannan (LAM) test for pulmonary TB and assessed the effect of a two-sample strategy.

**Methods:**

HIV-infected adults eligible for antiretroviral therapy were prospectively enrolled from Korle-Bu Teaching Hospital in Ghana and followed for minimum 6 months. We applied the LAM test on urine collected as a spot and early morning sample. Diagnostic accuracy was analysed for a microbiological TB reference standard based on sputum culture and Gene Xpert MTB/RIF results and for a composite reference standard including clinical follow-up data. Performance of sputum smear microscopy was included for comparison.

**Results:**

Of 469 patients investigated for TB, the LAM test correctly identified 24/55 (44 %) of microbiologically confirmed TB cases. Sensitivity of the LAM test was positively associated with hospitalisation (67 %), Modified Early Warning Score > 4 (57 %) and subsequent death (71 %). LAM test specificity was 95 % increasing to 98 % for the composite reference standard. A two-sample LAM test strategy did not improve test performance. Using concentrated sputum for Ziehl-Neelsen and fluorescence microscopy in combination yielded a sensitivity of 31/55 (56 %) that increased to 35/55 (64 %) when the LAM test was added. Surprisingly, nontuberculous mycobacteria were cultured in 34/469 (7 %) and associated with a positive LAM test (*p* = 0.008).

**Conclusions:**

LAM test sensitivity was highest in patients with poor prognosis and subsequent death and did not increase with a two-sample strategy. A rigorous sputum microscopy strategy had superior sensitivity, but the simplicity of the LAM test holds operational possibilities as a TB screening method among severely sick patients.

**Electronic supplementary material:**

The online version of this article (doi:10.1186/s12879-015-1151-1) contains supplementary material, which is available to authorized users.

## Background

Tuberculosis (TB) is the leading cause of death among HIV-infected individuals initiating antiretroviral therapy (ART) in sub-Saharan Africa [1]. Undiagnosed TB remains highly prevalent in this population as identified in studies of intensified TB screening pre-ART [2–4]; the highest TB diagnostic yield achieved by microbiological screening of all HIV-infected individuals regardless of presenting symptoms [5]. While culture based diagnostics remain the gold standard for TB diagnosis, it is time-consuming and unavailable in many TB endemic settings. The fully automated GeneXpert®MTB/RIF (“Xpert”) assay can provide diagnosis within 2 hours, but wide implementation of this assay is impeded by high costs and requirements for electricity and maintenance [6, 7]. Often, TB diagnosis relies on symptom-based screening, chest x-ray and sputum smear microscopy for Acid Fast Bacilli (AFB) although these tools have demonstrated limited performance [3, 8–10]. Recently, the DETERMINE TB-LAM Ag test (“LAM test” Alere, Waltham, MA, USA) was commercialised for point-of-care TB diagnosis. It is a lateral flow immunochromatographic strip test developed to detect the mycobacterial lipoarabinomannan (LAM) antigen released from metabolically active or degrading mycobacteria [11]. It can be applied directly on urine and provides a result within 30 minutes. Previous LAM performance studies have reported great variation in test sensitivity and specificity [12–19]. A consensus paper on the LAM test highlights issues with its performance and appropriate target group and calls for further evaluations of the LAM test in diverse settings [20].

We undertook a prospective study in Ghana to evaluate the diagnostic accuracy of the LAM test to diagnose pulmonary TB among HIV-infected individuals eligible for ART. We assessed a two-sample strategy, similar to what is conventionally applied for sputum microscopy, and explored the trade-off between the increase in sensitivity against reduction in specificity when using the LAM test as an add-on test to sputum smear microscopy.

## Methods

### Design

This is a cross sectional diagnostic accuracy study with a longitudinal follow-up of minimum 6 months.

### Study setting and population

As part of the DETECT HIV-TB study, participants were recruited prospectively between January 2013 and March 2014 from the out- and inpatient departments, Fevers Unit, Korle-Bu Teaching Hospital; Ghana’s largest public hospital situated in the capital city Accra. The Fevers Unit provides ART services including adherence counselling, medical care and laboratory services for HIV-infected individuals. The overall HIV-epidemic in Ghana is moderate with a prevalence of 1.4 % in the general population [21]. In 2013, the TB Control Programme in Ghana conducted a national TB prevalence survey that found an overall adult TB prevalence of 356/100,000 (personal communication with the programme manager for the National TB Control Programme, Ghana, unpublished data). This is much higher than the WHO estimated prevalence of 71/100,000 and questions the estimated case detection rates of 88 %, which is likely to be an overestimation [22].

HIV-infected adults were consecutively enrolled into the study if ≥18 years and eligible for lifelong ART, i.e. severe and advanced HIV clinical disease (WHO clinical stage 3 or 4 disease), a blood CD4 cell count ≤350 cells/μl or pregnant [23, 24]. Patients on antituberculous treatment of more than 2 days within the last 3 months before enrolment or who were unable to produce sputum or urine samples were excluded. Presence of TB related symptoms was not used as inclusion or exclusion criteria.

We used a standardized questionnaire to record demographic and clinical details including vital measures, height and weight, TB specific signs and symptoms including the WHO symptom screen with presence of more than one of the following symptoms: cough, fever, weight loss or night sweats [25] and previous history of TB. We calculated the Modified Early Warning Score (MEWS) for each participant as an indicator for illness severity. MEWS is a scoring system based on the level of deranged physiological parameters including systolic blood pressure, pulse rate, respiratory rate, body temperature and level of consciousness. A cut-off score > 4 has been considered predictive of poor outcomes [26]. Blood CD4 cell count was obtained for participants at enrolment. Full blood count and x-ray interpretation was obtained if available through routine laboratory investigations.

At enrolment, participants were requested to provide a spot urine specimen for LAM testing and one respiratory specimen for sputum smear microscopy, mycobacterial culture and Xpert assay. Participants were further asked to deliver an early morning urine and sputum sample within 7 days after enrolment. Medical records were reviewed from time of enrolment to a minimum of 6 months post-enrolment. If medical records were unavailable, an interview with the participant or relatives of the participant was conducted by phone. The following were recorded: vital status, lost to follow-up, transfer out, start on antituberculous treatment.

### Ethics

Informed consent was obtained in writing from each participant prior to enrolment in the study. The study protocol was approved by the Ethical and Protocol Review Committee, University of Ghana Medical School (MS-Et./M.4–P 3.3/2012-13) and evaluated by the Developing Country Committee of the Danish National Committee on Health Research Ethics (No. 1302133/1206169). Urine LAM test results were not used for treatment decision-making, but sputum microscopy, culture and Xpert results were communicated to the responsible clinicians.

We followed the Standards for the Reporting of Diagnostic accuracy studies (STARD) criteria [27]. Additional file 1 displays the STARD checklist (For more on STARD see http://www.stard-statement.org/).

### Urine sample analysis

Urine samples were transported to the Department of Medical Microbiology within Korle-Bu Teaching Hospital premises where study staff applied the LAM test on fresh urine in accordance with the manufacturer’s instructions. A 60 μL of unprocessed urine was applied to the sample pad at the bottom of the test strip. The test result was read between 25–35 minutes later and graded by comparing the test strip with a reference card. We used the original 2012 reference scale card that consisted of 5 colour intensity grades. The test band was graded as zero if no visual band appeared and graded 1 through 5 for a visualized band of equal intensity as those on the reference card. If a faint band was observed with intensity lower than the grade 1 cut-point it was recorded as “faint”. Additional file 2 provides a figure of the reference card used and its interpretation. Each test was graded by two individual readers blinded to their counterpart’s observations and to the patient identity through the use of anonymous study ID’s. Reference standard results were not known at the time of LAM testing.

### Sputum sample analysis

Sputum samples were analysed at the Reference TB Laboratory at Noguchi Memorial Institute for Medical Research or the TB laboratory at Korle-Bu Teaching Hospital. Samples were processed according to standardized protocols for mycobacterial microscopy and culture by trained laboratory technicians [28, 29]. Sputum samples were decontaminated with N-acetyl-L-cysteine and sodium hydroxide. Smears of centrifuged sputum sediment were examined microscopically and graded for AFB using both Ziehl-Neelsen (ZN) staining method and fluorescence microscopy of auramine O stained smears. After re-suspension in phosphate buffer the sputum sediment was cultured for mycobacteria using both solid Lowenstein-Jensen medium and the BACTEC mycobacteria growth indicator tube (MGIT) 960 system (BD Diagnostics, Sparks, MD, USA) and incubated for up to 8 and 6 weeks respectively. Positive cultures were re-assessed for AFBs using ZN smear microscopy and an anti-MPB64 antibody assay was used on AFB positive cultures to confirm presence of *M. tuberculosis.* The GeneXpert MTB-RIF assay was performed on either fresh sputum sample or on sputum sediment according to the manufacturer’s specifications (Cepheid, CA, USA) after it became available at the Chest Clinic TB laboratory to also confirm TB.

### Diagnostic classification for analysis

We defined a positive LAM test result as a test band with intensity equal to or greater than the grade 2 cut-point [14]. The first reading of the first sample was considered the study result and used for all data analysis except for analysis of inter-reader agreement and accuracy of a two-sample strategy.

In the absence of a single suitable reference standard for TB diagnosis in a population of severely immunocompromised HIV-infected individuals we used the following composite TB case definition to categorize participants:

“Confirmed TB” if *M. tuberculosis* culture positive or Xpert positive in any of the sputum samples.

“Possible TB” if no positive culture or Xpert results for TB, but one of the following; sputum smear microscopy positive i.e. smears graded as scanty, 1+, 2+, and 3+; a clinical-radiological picture highly suggestive of TB and started on antituberculous treatment within two months; a clinical diagnosis of active TB by a non-study clinician and started on treatment within two months; death within two months of enrolment reported to be due to TB per medical record.

“Non–TB” if not meeting criteria for “Confirmed” or “Possible” TB. Participants with growth of nontuberculous mycobacteria (NTM) and no positive cultures or Xpert results for *M. tuberculosis* were assigned to this group.

### Statistical analysis

**Fig. 1:**
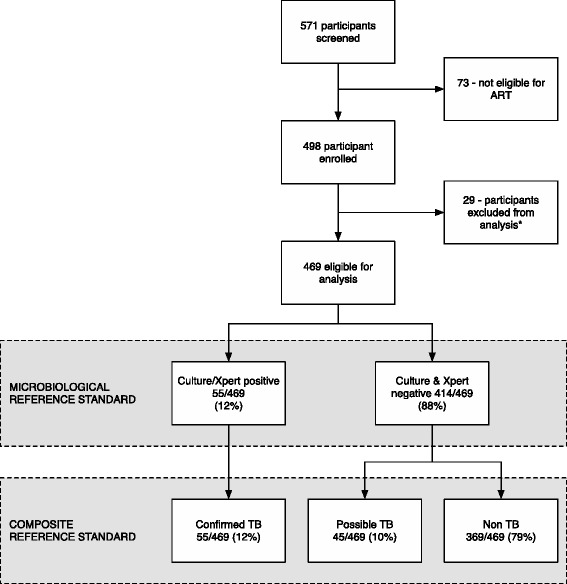
Flowchart of study participants and analysis. *Of 29 participants excluded; three (3) were on antituberculous treatment; twenty one (21) had no sputum samples; and five (5) had no urine sample. The remaining 469 participants were eligible for analysis with at least 1 sputum and 1 urine sample available

Descriptive analysis was used to characterize the study population and reported with interquartile range (IQR) and standard deviations (SD) as appropriate. Kappa statistics were used to determine inter-reader agreement between LAM test results and agreement between test results reported with the standard error (SE). Accuracy measures (sensitivity, specificity, positive predictive values (PPV), negative predictive values (NPV) and likelihood ratio (LR)) were calculated with 95 % Confidence Interval (CI). In our primary analysis, we used a microbiological reference standard comparing “Confirmed TB” vs. participants with no positive cultures or positive Xpert results. In the secondary analysis we used a composite reference standard for TB and combined “Confirmed TB” and “Possible TB” for calculation of sensitivities versus “Non TB” cases to calculate specificity. Figure 1 outlines the analysis of groups. For subgroup analysis we stratified participants by: enrolment site (hospitalised patients vs. outpatients); CD4 cell count (CD4 < 100 cells/mm^3^ vs. CD4 ≥ 100 cells/mm^3^); MEWS (MEWS > 4 vs. MEWS ≤ 4); and vital status at 2 months (dead vs. alive). Sensitivity and specificity was compared across strata using chi-square test or Fisher Exact test as appropriate. We determined diagnostic accuracy for LAM test in combinations with sputum smear microscopy and for the two-sample LAM test strategy. When assessing performance of a combination of tests, the result was considered positive if any of the tests were positive. The result was considered negative if both tests were negative. McNemar’s test was used to compare two different test sensitivities and specificities. The cumulative probabilities of death were estimated by means of the Kaplain-Meier method, compared according to LAM test results with the log-rank test. Statistical significance was defined as a two-sided p-value less than 0.05 and all analysis were conducted using STATA™ version 13.1 software.

## Results

### Participants

In the study period, 571 HIV-infected adults were screened and 469 were eligible according to our inclusion criteria (Fig. 1). In total, 399 (85 %) were enrolled from the outpatient clinic and 70 (15 %) were hospitalised patients (Table 1). Participants produced a mean of 1.8 (SD 0.36) urine samples with two urine samples obtained from 396 (84 %) participants. Two sputum samples were collected from 371 (79 %) participants with a mean of 1.8 (SD 0.41) per participant.

**Table 1:** Characteristics of study population

	All	Confirmed TB	Possible TB	Non-TB
	*n* = 469	*n* = 55	*n* = 45	*n* = 369
Enrolment site				
Out patients	399 (85 %)	40 (73 %)	20 (44 %)	339 (92 %)
In patients	70 (15 %)	15 (27 %)	25 (56 %)	30 (8 %)
Median Age in years (IQR)	38 (31–45)	37 (29–44)	37 (33–41)	38 (32–45)
Female	301 (64 %)	24 (44 %)	29 (64 %)	241 (65 %)
Median CD4 (IQR)*	127 (35–256)	92 (41–176)	46 (12–176)	142 (41–282)
CD4 < 100	195 (43 %)	29 (55 %)	28 (65 %)	138 (39 %)
CD4 > =100	259 (57 %)	24 (45 %)	15 (35 %)	220 (61 %)
Mean MEWS (SD)	3 (1.95)	5 (2.20)	4 (2.37)	3 (1.59)
MEWS > 4	101 (22 %)	35 (63 %)	19 (42 %)	47 (13 %)
MEWS < = 4	368 (78 %)	20 (36 %)	26 (58 %)	322 (87 %)
Median HGB g/dl** (IQR)	9.7 (8.1-11.2)	8.2 (6.2-9.6)	8.0 (6–10)	10 (9–12)
Median BMI (IQR)	20 (18–22)	18 (16–19)	19 (16–21)	21 (18–23)
TB suspects, WHO symptom screen	426 (91 %)	53 (96 %)	43 (96 %)	330 (90 %)
Current Cough	222 (48)	42 (76 %)	28 (62 %)	152 (41 %)
Fever	238 (52 %)	45 (82 %)	28 (62 %)	165 (45 %)
Weight loss	390 (83 %)	51 (93 %)	39 (87 %)	300 (81 %)
Night Sweats	161 (34 %)	26 (49 %)	16 (36 %)	119 (32 %)
Previous history of TB	28 (6 %)	4 (7 %)	2 (4 %)	22 (6 %)
Overall mortality***	81 (17 %)	18 (33 %)	23 (51 %)	40 (11 %)

*CD4 count missing for 15 participants

**HGB missing for 39 participants

***80 participants were lost to follow-up at 6 months and 11 transferred out

*IQR* = Interquartile range, *SD* = Standard Deviation

TB was confirmed bacteriologically in 55 cases (prevalence 11.7 %; 95 % CI 9.1-15.0) and additional 45 cases were defined as “Possible TB” (prevalence 9.6 %; 95 % CI 7.2-12.6). Sputum microscopy for AFB was positive in 31/55 (56 %) of “Confirmed TB” cases. Among the 45 “Possible TB” cases, 6/45 (13 %) were categorised as “Possible TB” based on a positive sputum microscopy for AFB, 25/45 (56 %) based on start-up of antituberculous treatment within 2 months, and 14/45 (31 %) died within 2 months of follow-up with TB stated as the cause of death.

### Urine LAM test performance

All LAM tests provided a valid result with visible control bar. The distribution of LAM test results across band intensity grades was: no band, 194 (41 %); faint band, 168 (36 %); grade 1, 62 (13 %); grade 2, 10 (2 %); grade 3, 10 (2 %); grade 4, 16 (4 %) and grade 5, 9 (2 %). Inter-rater agreement between the readers as to presence versus absence of a LAM test band with intensity of grade 2 cut-point was 99.1 % (kappa 0.94; SE 0.05). Agreement as to test band grade was 69.2 % (kappa 0.54; SE 0.03) mainly due to inter-rater variability between recording of grade 1 cut-point intensity vs. faint. Additional file 3a + b details the inter-rater variability.

#### Analysis 1–Microbiological reference standard

For grade 2 cut-point positivity threshold the urine LAM test had an overall diagnostic sensitivity of 44 % (95 % CI; 30–58) and specificity of 95 % (95 % CI; 92–97) using the microbiological reference standard (Table 2). Additional file 4 provides a table with the diagnostic accuracy of the LAM test by all grade cut-point. Additional file 5 displays the Receiver Operator Characteristic (ROC) curve for the LAM test. In the subgroup analysis (Table 3), LAM test sensitivity increased significantly among hospitalised patients (*p* = 0.035), participants with MEWS > 4 (*p* = 0.008) and in participants who died within 2 months of follow-up (*p* = 0.013). The increase in sensitivity among participants with low CD4 did not reach statistically significance. The inverse pattern was seen for test specificity being significantly lower among strata with higher degree of diseases severity or death as outcome.

**Table 2:** Overall performance of LAM test for diagnosis of tuberculosis

	Total	Sensitivity		Specificity		LR (+)	LR (−)	PPV	NPV
		N	% (95 % CI)	N	% (95 % CI)	(95 % CI)	(95 % CI)	% (95 % CI)	% (95 % CI)
LAM test overall									
Microbiological reference standard*	469	24/55	44 (30–58)	393/414	95 (92–97)	8.6 (5.1-14.4)	0.6 (0.5-0.8)	53 (38–68)	93 (90–95)
Composite reference standard**	469	36/100	36 (27–46)	360/369	98 (95–99)	14.8 (7.4-29.6)	0.7 (0.6-0.8)	80 (65–90)	85 (81–88)

*Primary analysis using a microbiological reference standard:” Confirmed TB” cases versus culture and Xpert negative cases

**Secondary analysis using a composite reference standard: “Confirmed TB” and “Possible TB” combined for calculations of sensitivity versus “Non TB”

*LR* (+) = Positive Likelihood Ratio, *LR* (−) = Negative Likelihood Ratio, *PPV* = Positive Predictive Value, *NPV* = Negative Predictive Value

**Table 3:** The overall sensitivity and specificity of the LAM test and stratified by subpopulation

LAM test overall			Total	Microbiological reference standard						Composite reference standard					
				Sensitivity			Specificity			Sensitivity			Specificity		
				N	% (95 % CI)	*p*-value	N	% (95 % CI)	*p*-value	N	% (95 % CI)	*p*-value	N	% (95 % CI)	*p*-value
			469	24/55	44 (30–58)		393/414	95 (92–97)		36/100	36 (27–46)		360/369	98 (95–99)	
	By Department		469												
		Inpatient	70	10/15	67 (38–88)		47/55	86 (73–94)		17/40	43 (27–59)		29/30	97 (83–100)	
		Out patient	399	14/40	35 (21–52)	**0.035**	346/359	96 (94–98)	**0.001**	19/60	32 (20–45)	0.269	331/339	98 (95–99)	0.74
	By CD4 strata*		454												
		CD4 < 100	195	14/29	48 (29–68)		148/166	89 (83–93)		24/57	42 (29–56)		130/138	94 (89–98)	
		CD4 > = 100	259	8/24	33 (16–55)	0.272	233/235	99 (97–100)	**<0.001**	9/39	23 (11–39)	0.054	219/220	100 (98–100)	**0.002**
	By MEWS		469												
		MEWS > 4	101	20/35	57 (39–74)		54/66	82 (70–90)		29/54	54 (40–67)		44/47	94 (83–99)	
		MEWS < = 4	368	4/20	20 (6–44)	**0.008**	339/348	97 (95–99)	**<0.001**	7/46	15 (6–29)	**<0.001**	316/322	98 (96–99)	0.061
	By Vital status at 60 days**		397												
		Dead	52	10/14	71 (42–92)		33/38	87 (72–96)		14/29	48 (29–68)		22/23	96 (78–100)	
		Alive	345	11/34	32 (17–51)	**0.013**	302/311	97 (95–99)	**<0.001**	14/58	24 (14–37)	**0.023**	281/287	98 (96–99)	0.483

*15 participants with missing values for CD4 were excluded from analysis

**63 participants were lost to follow-up at 2 months and 7 transferred out

*MEWS* = Modified Early Warning Score

#### Analysis 2–Composite reference standard for TB

When using the composite reference standard for TB, the overall LAM test specificity increased to 98 % (95 % CI; 95–99), but sensitivity reduced to 36 % (95 % CI; 27–46) (Table 2). Sensitivity increased for the strata with poor outcome, while specificity remained above 94 % for all strata (Table 3).

### LAM test performance with a two-sample strategy using the microbiological reference standard

Of all participants, 396/469 (84 %) was able to deliver two urine samples for LAM test. Among these, the LAM test correctly identified 14/40 (35 %) of the “Confirmed TB” cases in the first sample tested and additionally 2 “Confirmed TB” cases 16/40 (40 %) when including positive LAM test results from the second sample. The additional two cases both had a grade 1 band intensity at the first LAM test. The increase in sensitivity was not statistically significant (35 % vs. 40 %; *p* = 0.5) and specificity dropped significantly from 96 % to 92 % (*p* < 0.001) when the second LAM test was applied. The LAM test was positive in 40/458 (9 %) of the spot samples and 45/407 (11 %) of the early morning samples. Agreement between spot and morning LAM test results was 95.5 % (kappa 0.72; SE 0.05). Additional file 6a + b detail the inter-variability between spot and morning samples. Sensitivity of urine LAM test performed on a spot sample was 40 % (21/52; 95 % CI: 27–55) slightly lower than when applied on an early morning urine samples with a sensitivity of 42 % (18/43; 95 % CI: 27–58). Specificity was 95 % (387/406; 95 % CI: 93–97) and 93 % (337/364; 95 % CI: 89–95) for spot and morning sample respectively.

### Sputum smear microscopy performance alone and in combination with LAM test using the microbiological reference standard

ZN microscopy detected 29/55 (53 %) of the confirmed TB cases and ZN + fluorescence microscopy in combination detected 31/55 (56 %) (Table 4). Concordance between LAM test and microscopy was moderate both for ZN microscopy alone (91.4 %; kappa 0.45; SE 0.05) and ZN + fluorescence microscopy (91.4 % kappa 0.47; SE 0.05). Similar to the LAM test accuracy, the sensitivity of sputum microscopy increased in subgroups with poor prognosis (data not shown). Combining LAM test to ZN and fluorescence microscopy maximised sensitivity to 35/55 (64 %) non-significantly higher than for the ZN and fluorescence microscopy alone. There were four additional TB cases detected by adding the LAM test; all had CD4 < 100 cells/mm^3^ and two of them subsequently died. The LAM false positive cases were 26 of whom 17 (65 %) were defined as “possible TB”.

**Table 4:** Sensitivity and specificity of the LAM test, sputum smear microscopy and combinations of the tests

	Total	Sensitivity		Specificity		LR (+)	LR (−)	PPV	NPV
		N	% (95 % CI)	N	% (95 % CI)	% (95 % CI)	% (95 % CI)	% (95 % CI)	% (95 % CI)
LAM test	469	24/55	44 (30–58)	393/414	95 (92–97)	8.6 (5.1-14.4)	0.6 (0.5-0.8)	53 (38–68)	93 (90–95)
ZN microscopy	467	29/55	53 (39–66)	406/412^a^	99 (97–100)	36.2 (15.7-83.3)	0.5 (0.4-0.6)	83 (66–93)	94 (91–96)
ZN + Fluorescence microscopy	467	31/55	56 (42–70)	406/412^a^	99 (97–100)	38.7 (16.9-88.5)	0.4 (0.3-0.6)	84 (68–94)	94 (92–96)
LAM + ZN microscopy	469	34/55	62 (48–75)^a^	388/414	94 (90–96)^a,b^	9.8 (6.4-15.1)	0.4 (0.3-06)	57 (43–69)	95 (92–97)
LAM + ZN + Fluorescence Microscopy	469	35/55	64 (50–76)^a^	388/414	94 (91–96)^a,c^	10.1 (6.6-15.5)	0.4 (0.3-0.6)	57 (44–70)	95 (93–97)

For combinations of test a positive result was recorded if any of the tests were positive

^a^ *p* < 0.05 for comparison with LAM test alone

^b^ *p* < 0.05 for comparison with ZN microscopy alone

^c^ *p* < 0.05 for comparison with ZN + Fluorescence microscopy

*LR* (+) = Positive Likelihood Ratio, *LR* (−) = Negative Likelihood Ratio, *PPV* = Positive Predictive Value, *NPV* = Negative Predictive Value, *ZN* = Ziehl Neelsen

### LAM test cross reactivity with nontuberculous mycobacteria

NTM was cultured in sputum from 34 (prevalence 7.2 %; 95 % CI 5.2-10.0) participants and additionally two participants were co-infected with NTM and TB. The LAM test was positive in 5/34 (15 %, 95 % CI; 5–31) participants with a positive LR+ of 1.6 (95 % CI; 0.7-3.8). Compared to those without any mycobacteria, a positive NTM culture was associated with a positive LAM test (4 % vs. 15 %; *p* = 0.008).

### Mortality

**Fig. 2:**
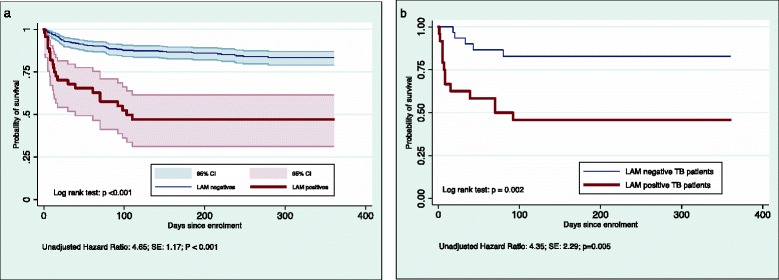
Cumulative probability of survival. **a** Shown for the full study population (n = 469) by LAM test status. **b** Shown for confirmed TB cases (n = 55) by LAM test status

During the study 81 (17 %) of the participants died after a median of 25 days from enrolment. The 6-month mortality was 73 (16 %), 80 (17 %) participants were lost to follow-up (LTFU) and 11 (2 %) transferred out. Confirmed TB patients had a 2-month mortality of 14/55 (25 %) and at 6-month 18 (33 %) cases had died, 7 cases (13 %) were LTFU and 1 (4 %) transferred out. LAM positive participants had a significantly higher probability of death compared to LAM negative in the overall population (*p* < 0.001) (Fig. 2a) and among “confirmed TB” (*p* = 0.002) (Fig. 2b). LTFU among LAM positive participants was not significantly higher than among LAM negative (6/45; 18 % vs. 74/424; 13 %, *p* = 0.5). In a sensitivity analysis assuming that all participants LTFU had died, the probability of death remained significantly higher for LAM-positive than LAM-negative participants overall (*p* < 0.001) and among confirmed TB cases (*p* = 0.044).

## Discussion

We evaluated the urine LAM strip test for TB diagnosis among ART eligible adults in Ghana where TB is reported as the most common AIDS defining event and cause of death [30]. We found that rapid urine LAM test could identify 44 % of confirmed TB cases with a specificity of 95 % and that a two-sample strategy for the LAM test did not improve sensitivity. The LAM test was easily performed and had a high inter-reader reliability. Sensitivity increased to 67 % among hospitalised patients and was associated with death and high clinical illness score (MEWS > 4); further, we found a tendency of increased sensitivity among patients with lower CD4 cell counts. Our findings affirm that sensitivity of the LAM test is highest for the sickest patients and those holding the greatest risk of dying. This is consistent with findings by Lawn et al. who found increased LAM test sensitivity among patients with CD4 cell count < 100 cells/mm^3^, CRP > 200 mg/L, severe anaemia (<8.0 g/dL), advanced symptoms and subsequent death [31, 32]. To our knowledge, four other studies have evaluated urine LAM test among HIV-infected individuals irrespective of the presence of TB symptoms [12–15] and four other studies among HIV-infected TB suspects [16–19]. The lowest overall LAM test sensitivity of 25 % as reported by Balcha et al. in a study that included HIV-infected individuals with CD4 < 350 regardless of TB symptoms [12]. The highest sensitivity of 50 % was reported for grade 2 cut-point in a study among hospitalised TB suspects [18]. LAM test sensitivity was consistently the highest among hospitalised populations and strata with lower CD4 cell count, suggesting that critically ill patients are the appropriate target group for LAM testing. No study has previously reported a correlation with MEWS although this could be a simple and objective alternative to identify the target group for LAM testing. MEWS was originally developed to detect critically ill patients at risk of catastrophic deterioration in high-income countries [26], but has since gained a wider application in medical wards and intensive care units to predict hospital admission and mortality also in resource limited settings [33, 34]. A great advantage of MEWS is that it is based on simple measures that are routinely collected as part of most clinical consultations. Our finding of a significantly higher risk of death for LAM-positive compared with LAM-negative TB patients further emphasises that the LAM test identify TB patients with the greatest clinical need.

Specificity of the LAM test is paramount for utility of LAM as a screening test. We found considerably lower specificity among subgroups with signs of critical illness or subsequent death than the target of 95 % for new point-of-care tests for TB diagnosis [35]. Using the composite reference standard, however, increased overall specificity to 98 % and to 94 % in all subgroups with poor outcome. This probably reflects the well-known limitations of sputum culture and Xpert to detect TB among severely sick HIV-infected individuals. We sought to optimize our microbiological reference standard by performing two sputum cultures on both solid and liquid media, and by adding Xpert results. Despite these efforts, the microbiological reference standard leads to underestimation of specificity. This is similar to findings by Peter et al. where LAM test specificity increased from 78 % to 94 % when the composite reference standard was used instead of a microbiological reference standard [18]. Another key factor for test specificity is the positivity threshold for the LAM test as shown in previous studies [13, 16, 18]. There is consensus of using grade 2 cut-point as positivity threshold and in 2014 the LAM test manufacturers changed the reference scale card omitting the band corresponding to grade 1. However, we report a large number of tests with band-intensity fainter than the original grade 2 cut-point as did another prospective LAM study [16]. It is important to acknowledge that such visible bands, although fainter than the positivity threshold, in a clinical setting could prompt clinicians to interpret the LAM test as positive and lead to over treatment.

LAM is also a component of the NTM cell wall [36], but the possibility of NTM causing false positive LAM test results has been sparsely addressed. In a cohort of cystic fibrosis patients we previously reported that 2/23 (8.7 %) of NTM infected patients were LAM-positive at a grade 2 cut-point increasing to 9/23 (39.1 %) for grade 1 cut-point [37]. In the context of HIV infected individuals, 10 NTM culture positive cases have previously been described to be LAM positive [15, 38–40]. We found a positive LAM test for 5/34 (15 %) of NTM culture positives. The prevalence of NTM culture positive was high in our study and associated with a positive LAM test. While this raises concern that NTM among HIV infected individuals affect LAM test specificity, its importance needs to be evaluated in studies applying appropriate case definitions for NTM disease.

A two-sample test strategy including an early morning sample has not been evaluated to date for the LAM test. We found that an additional sample did not increase overall performance of the LAM test and agreement between results for a spot and morning sample was high. A recent study explored a two-test strategy performed on the same sample and did not show any added diagnostic value of the second test [13].

Previous studies found that the LAM test sensitivity was superior to sputum smear microscopy and found incremental diagnostic value of combining the two tests [12, 16, 19]. We found higher sputum smear microscopy sensitivity that contrasts other studies comparing microscopy with the LAM test. This could be due to our use of sputum concentration and fluorescence microscopy both known to increase diagnostic sensitivity [41, 42], and collection of two samples for the majority of participants. A similar and rigorous sputum sampling and microscopy methodology is not always achievable in a routine clinical setting and the LAM test could be preferred for reasons other than a higher diagnostic accuracy alone.

The LAM test is simple to perform at the bedside and collection of urine is simple and poses minimal biohazard; all attractive features in settings with overburdened TB laboratories. Urine based diagnosis further holds a potential to improve TB diagnosis in children with one study of LAM test showing reasonable sensitivity in HIV-positive TB infected children [43]. A number of other biomarkers have been detected at increased levels in the urine from patients with active TB [44, 45]. LAM remains among the most promising so far, but is limited by its modest sensitivity and use among HIV-infected individuals only.

The strengths of this study are prospective data collection, LAM testing on fresh urine and a minimum of 6 months’ follow-up. We chose to enrol HIV-infected individuals initiating ART regardless of clinical presentation as this target group has been identified as a particularly vulnerable group for prevalent and undiagnosed TB. We had several limitations with regard to our reference standard that did not include Xpert results for all participants or investigation of extrapulmonary samples that could have increased specificity further, especially among those with more advanced immunodeficiency. Moreover, we did not have capacity to perform sputum induction to improved sputum sample quality. Despite active follow-up through personal calls to participants and their relatives the LTFU was high, but comparable to several other African HIV-cohort studies [46]. Mortality is a frequent cause of LTFU [47, 48] and mortality rates in our study could have been higher had we performed a more thorough follow-up with e.g. house-visit. However, sensitivity analysis assuming that all participants LTFU had died did not change the association between LAM-positivity and mortality.

## Conclusions

Despite modest sensitivity, we found that the LAM test has a potential to improve TB case detection when applied as a screening test among the sickest patients and those at the greatest risk of dying, especially in settings without easy access to high quality microscopy, culture or more advanced diagnostic technologies. A two-sample strategy did not improve test performance.

## Additional files

Additional file 1:
**The STARD checklist.** (PDF 302 kb) Additional file 2:
**The original 2012 LAM test reference scale card used in the study and interpretation.** The integers under the reference card correspond to grade 1 cut-point (1), grade 2 cut-point (2), grade 3 cut-point (3), grade 4 cut-point (4) and grade 5 cut-point (5). *Interpretation of positivity threshold is indicated by “Considered positive” (PDF 4536 kb) Additional file 3:
**Inter-rater variability for reader 1 and 2.** (a.) Shown for presence versus absence of a test band with intensity grade 2 cut-point or higher (b.) Shown by grade cut-points (PDF 57 kb) Additional file 4:
**Accuracy of the LAM test by all grade cut-points.** (PDF 33 kb) Additional file 5:
**Receiver operator characteristic curve for the LAM test.** The integers adjacent to points on the graph correspond to faint (F), grade 1, grade 2, grade 3, grade 4 and grade 5 cut-point (PDF 42 kb) Additional file 6:
**Inter-variability for spot and morning samples.** (a.) Shown for presence versus absence of a test band with intensity grade 2 cut-point or higher (b.) Shown by test band grade cut-points (PDF 56 kb)

## Abbreviations

AFB: Acid fast bacilli
ART: Antiretroviral therapy
FM: Fluorescence microscopy
IQR: Interquartile range
LAM: Lipoarabinomannan
LR: Likelihood ratio
MEWS: Modifies early warning score
MTB/RIF: Mycobacterium tuberculosis/resistance to rifampicin
NPV: Negative Predictive Value
NTM: Nontuberculous mycobacteria
PPV: Positive Predictive Value
TB: Tuberculosis
WHO: World Health Organization
ZN: Ziehl-Neelsen
SD: Standard Deviation
SE: Standard Error
