# Supplementary material for: Diagnostic accuracy of the rapid urine lipoarabinomannan test for pulmonary tuberculosis among HIV-infected adults in Ghana–findings from the DETECT HIV-TB study
Source: BMC Infect Dis. 2015 Oct 1;15:407. doi: 10.1186/s12879-015-1151-1 (PMC4591579; doi:10.1186/s12879-015-1151-1)
Supplement: Additional file 2: — The original 2012 LAM test reference scale card used in the study and interpretation. The integers under the reference card correspond to grade 1 cut-point (1), grade 2 cut-point (2), grade 3 cut-point (3), grade 4 cut-point (4) and grade 5 cut-point (5). *Interpretation of positivity threshold is indicated by “Considered positive” (PDF 4536 kb) [file 12879_2015_1151_MOESM2_ESM.pdf]

Additional file 2: The original 2012 LAM test reference scale card used in the study and interpretation. The integers under the reference card correspond to grade 1 cut-point (1), grade 2 cut-point (2), grade 3 cut-point (3), grade 4 cut-point (4) and grade 5 cut-point (5).

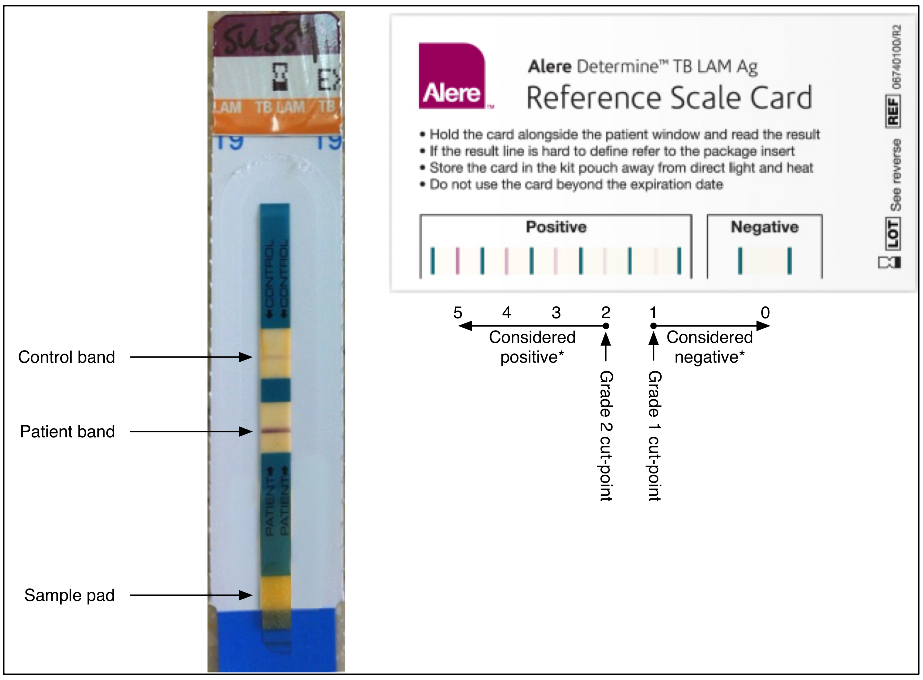

*Interpretation of positivity threshold is indicated by "Considered positive".
