# Supplementary material for: Diagnostic accuracy of the rapid urine lipoarabinomannan test for pulmonary tuberculosis among HIV-infected adults in Ghana–findings from the DETECT HIV-TB study
Source: BMC Infect Dis. 2015 Oct 1;15:407. doi: 10.1186/s12879-015-1151-1 (PMC4591579; doi:10.1186/s12879-015-1151-1)
Supplement: Additional file 3: — Inter-rater variability for reader 1 and 2. (a.) Shown for presence versus absence of a test band with intensity grade 2 cut-point or higher (b.) Shown by grade cut-points (PDF 57 kb) [file 12879_2015_1151_MOESM3_ESM.pdf]

**Additional file 3a: Inter-rater variability for reader 1 and 2 as to presence versus absence of a test band with intensity grade 2 cut-point or higher**			
	**Reader 2**		
**Reader 1**	**0**	**1**	**Total***
**0**	398	4	402
**1**	0	39	39
**Total**	398	43	441

Agreement as to test band grade 2 or higher was 99.1% (kappa 0.94; SE 0.05)

**Additional file 3b: Inter-rater variability for reader 1 and 2 by test band grade cut-points**								
	**Reader 2**							
**Reader 1**	**0**	**Faint**	**1**	**2**	**3**	**4**	**5**	**Total***
**0**	165	9	8	0	0	0	0	182
**Faint**	63	78	17	1	0	0	0	159
**1**	11	16	31	3	0	0	0	61
**2**	0	0	0	8	0	1	0	9
**3**	0	0	0	1	7	1	0	9
**4**	0	0	0	0	0	10	3	13
**5**	0	0	0	0	0	2	6	8
**Total**	239	103	56	13	7	14	9	441

Agreement as to test band grade was 69.2% (kappa 0.54; SE 0.03)

* For 28 participants the results of the LAM test for the same spot urine sample was not read by both raters why the total is less than the full study population of 469 participants
