# Supplementary material for: Diagnostic accuracy of the rapid urine lipoarabinomannan test for pulmonary tuberculosis among HIV-infected adults in Ghana–findings from the DETECT HIV-TB study
Source: BMC Infect Dis. 2015 Oct 1;15:407. doi: 10.1186/s12879-015-1151-1 (PMC4591579; doi:10.1186/s12879-015-1151-1)
Supplement: Additional file 4: — Accuracy of the LAM test by all grade cut-points. (PDF 33 kb) [file 12879_2015_1151_MOESM4_ESM.pdf]

Additional file 4. Accuracy of the LAM test by by band grade cut-point

	Total N (%)	Sensitivity		Specificity		LR (+)	LR (-)	PPV	NPV
**Test band grade**		**N**	**% (95% CI)**	**N**	**% (95% CI)**	**% (95% CI)**	**% (95% CI)**	**% (95% CI)**	**% (95% CI)**
**≥ Faint**	168 (36%)	47/55	85 (73-93)	186/414	45 (40-50)	1.5 (1.3-1.8)	0.3 (0.2-0.6)	17 (13-22)	96 (92-98)
**≥ Grade 1 cut-point**	62 (13%)	34/55	62 (48-75)	341/414	82 (78-86)	3.5 (2.6-4.7)	0.5 (0.3-0.7)	32 (23-42)	94 (91-96)
**≥ Grade 2 cut-point**	10 (2%)	24/55	44 (30-58)	393/414	95 (92-97)	8.6 (5.1-14.4)	0.6 (0.5-0.8)	53 (38-68)	93 (90-95)
**≥ Grade 3 cut-point**	10 (2%)	34/55	38 (25-52)	400/414	97 (94-98)	11.3 (6.1-20.9)	0.6 (0.5-0.8)	60 (42-76)	92 (89-95)
**≥ Grade 4 cut-point**	16 (4%)	40/55	27 (16-41)	404/414	98 (96-99)	11.3 (5.3-23.9)	0.7 (0.6-0.9)	60 (39-79)	91 (88-94)
**≥ Grade 5 cut-point**	9 (2%)	51/55	7 (2-18)	409/414	99 (97-100)	6 (1.7-21.8)	0.9 (0.9-1)	44 (14-79)	89 (86-92)

LR (+) = Positive Likelihood Ratio; LR (-) = Negative Likelihood Ratio; PPV = Positive Predictive Value; NPV = Negative Predictive Value
