# Supplementary material for: Diagnostic accuracy of the rapid urine lipoarabinomannan test for pulmonary tuberculosis among HIV-infected adults in Ghana–findings from the DETECT HIV-TB study
Source: BMC Infect Dis. 2015 Oct 1;15:407. doi: 10.1186/s12879-015-1151-1 (PMC4591579; doi:10.1186/s12879-015-1151-1)

Additional file 5: Receiver operator characteristic curve for the LAM test.
The integers adjacent to points on the graph correspond to faint (F), grade 1, grade 2, grade 3, grade 4 and grade 5.

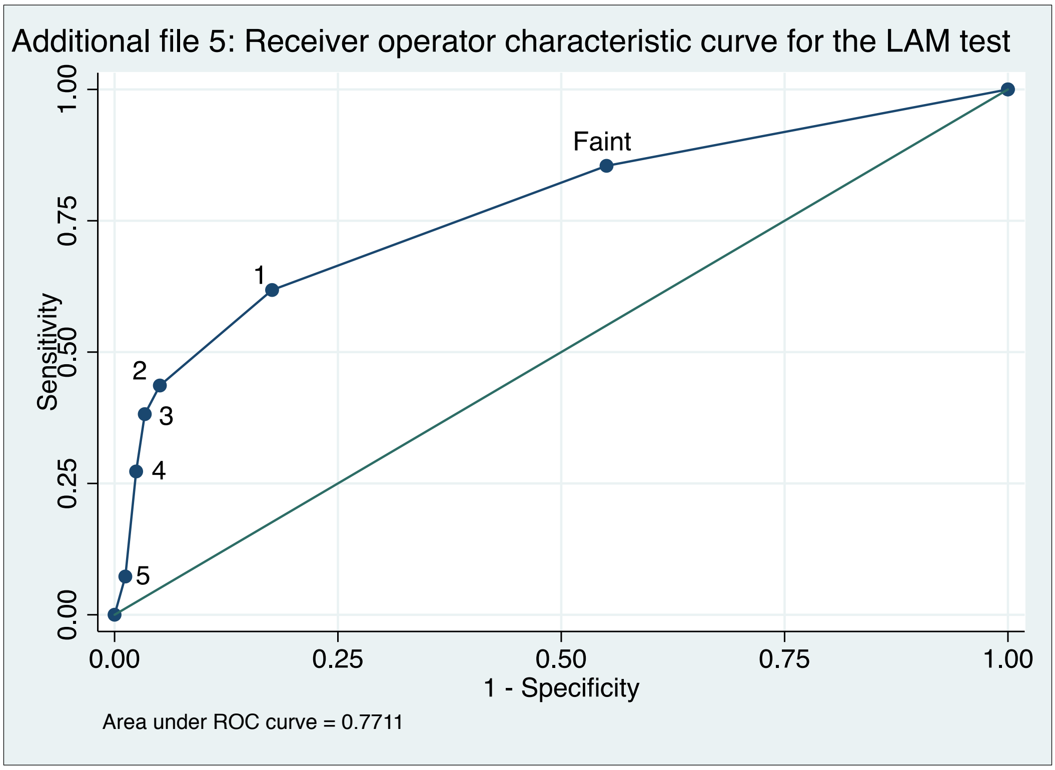

Supplement: Additional file 5: — Receiver operator characteristic curve for the LAM test. The integers adjacent to points on the graph correspond to faint (F), grade 1, grade 2, grade 3, grade 4 and grade 5 cut-point (PDF 42 kb) [file 12879_2015_1151_MOESM5_ESM.pdf]
