# Supplementary material for: Diagnostic accuracy of the rapid urine lipoarabinomannan test for pulmonary tuberculosis among HIV-infected adults in Ghana–findings from the DETECT HIV-TB study
Source: BMC Infect Dis. 2015 Oct 1;15:407. doi: 10.1186/s12879-015-1151-1 (PMC4591579; doi:10.1186/s12879-015-1151-1)
Supplement: Additional file 6: — Inter-variability for spot and morning samples. (a.) Shown for presence versus absence of a test band with intensity grade 2 cut-point or higher (b.) Shown by test band grade cut-points (PDF 56 kb) [file 12879_2015_1151_MOESM6_ESM.pdf]

Additional file 6a: Inter-variability for spot and morning samples as to presence versus absence of a test band with intensity grade 2 cut-point or higher

Spot	Morning 0	1	Total*
0	352	14	366
1	4	26	30
Total	356	40	396

Agreement as to test band grade 2 or higher was 95.5% (kappa 0.72; SE 0.05)

Additional file 6b: Inter-variability for spot and morning samples by test band grade cut-points

Spot	Morning 0	Faint	1	2	3	4	5	Total*
**0**	72	60	26	2	3	2	0	165
**Faint**	33	88	22	2	0	1	1	147
**1**	14	8	29	3	0	0	0	54
**2**	0	1	2	3	2	1	0	9
**3**	0	0	0	4	1	1	0	6
**4**	1	0	0	1	0	5	3	10
**5**	0	0	0	0	0	1	4	5
**Total**	120	157	79	15	6	11	8	396

Agreement as to test band grade was 51.0% (kappa 0.30; SE 0.03)

* For 73 (15.6%) participants we did not have both spot and morning urine samples available
